# Court applications for withdrawal of artificial nutrition and hydration from patients in a permanent vegetative state: family experiences

**DOI:** 10.1136/medethics-2015-102777

**Published:** 2015-10-20

**Authors:** Celia Kitzinger, Jenny Kitzinger

**Affiliations:** 1 Department of Sociology, University of York, York, UK; 2 School of Journalism, Culture & Media Studies, Cardiff University, Cardiff, UK

**Keywords:** Family, Right to Refuse Treatment, Public Law, Prolongation of Life and Euthanasia, Legal Aspects

## Abstract

Withdrawal of artificially delivered nutrition and hydration (ANH) from patients in a permanent vegetative state (PVS) requires judicial approval in England and Wales, even when families and healthcare professionals agree that withdrawal is in the patient's best interests. Part of the rationale underpinning the original recommendation for such court approval was the reassurance of patients’ families, but there has been no research as to whether or not family members are reassured by the requirement for court proceedings or how they experience the process. The research reported here draws on in-depth narrative interviews with 10 family members (from five different families) of PVS patients who have been the subject of court proceedings for ANH-withdrawal. We analyse the empirical evidence to understand how family members perceive and experience the process of applying to the courts for ANH-withdrawal and consider the ethical and practice implications of our findings. Our analysis of family experience supports arguments grounded in economic and legal analysis that court approval should no longer be required. We conclude with some suggestions for how we might develop other more efficient, just and humane mechanisms for reviewing best interests decisions about ANH-withdrawal from these patients.

## Introduction

In *Airedale NHS Trust v Bland*,^1^ the House of Lords established a lasting precedent that it is not in the best interests of a patient in a permanent vegetative state (PVS) to receive artificial nutrition and hydration (ANH) and that it can be lawful to withdraw it. The case prompted a great deal of subsequent ethical analysis about sanctity of life, quality of life, whether a permanently unconscious person has ‘interests’ and if so whether it can ever be in their best interests to die, the definition of acts and omissions, ‘medical treatment’ and ‘basic care’.^2–4^ This article addresses one aspect of an issue that has been much less discussed—family experiences of the continuing requirement, in England and Wales, for judicial approval before ANH can lawfully be withdrawn from PVS patients.

The vast majority of decisions to withdraw or withhold life-sustaining medical treatments are never referred to court but are decided and implemented on the basis of common law and professional guidelines.^5^ ^6^ These include stipulations that doctors should not administer ‘futile’ or ‘burdensome’ treatments, or impose treatments that are refused by a capacitous patient or are not in the best interests of an incapacitous patient. Under any such circumstances, it is in accordance with the law and with professional ethics to withhold or withdraw life-sustaining treatments, including ANH, without legal review. The situation for PVS patients (in England and Wales) is anomalous in that withdrawing or withholding ANH from this patient group requires legal review—even in straightforward cases when families and healthcare teams agree that cessation of ANH is in the patient's best interests.

The requirement for legal review for withdrawing or withholding ANH from patients in PVS is a legacy of *Bland* in which the House of Lords recommended that a court declaration should be required in such cases ‘at least for the time being and until a body of experience and practice has been built up which might obviate the need for application in every case’.^i^ Despite the view expressed by the British Medical Association,^7^ more than a decade after *Bland*, that PVS cases should no longer inevitably require court review, it is now a legal obligation governed by Practice Direction 9E issued under the authority of the Court of Protection Rules 2007 (S.I. 2007/1744). Continuing judicial involvement in ANH-withdrawal from PVS patients has been criticised for various reasons. It is out of kilter with international law^8^ ^9^ and inconsistent with various aspects of domestic law.^9–11^ It is also expensive (about £122 000 per patient)^12^ and is implicated in a delay (on average 9 months) between the point at which a best interests decision is made to withdraw ANH and the time at which that decision can be lawfully implemented.^9^ This article adds to this body of concern by focusing specifically on the experience of the families of these patients.

Part of the rationale underpinning the original House of Lords’ recommendation in 1993 for court approval in PVS cases was ‘the reassurance of patients’ families’.^ii^ This is despite the fact that the parents in *Bland,* who supported withdrawal of ANH from their son, ‘could not understand why the courts should be involved’ and were reluctant to give their approval for a court application.^13^ There was no research at the time (nor has there been since) on whether or not family members in general are ‘reassured’ by the requirement for court proceedings—or more generally on how they experience the process. In the research reported here, we address this gap, analysing empirical evidence to understand how family members perceive and experience the process of applying to the courts for ANH-withdrawal and considering the ethical and practice implications of our findings.

## Method

The research reported here arose out of a broader in-depth interview study of families in the UK with experience of having a relative in a vegetative or minimally conscious state. More information about the methodology of the broader study has already been published^14^ as have findings relating to families’ perspectives on a range of different aspects of their experience, including (eg) medical decision making,^15^ physiotherapy^16^ and functional magnetic resonance imaging (fMRI) scanning.^17^

In an earlier article in this journal,^14^ we explored family perspectives on withdrawal of ANH. We focused on the powerful symbolism associated with food and water, and our data showed that very few families of PVS or minimally conscious state (MCS) patients—even those who have come to believe that the patient would rather die than continue in their current state—considered it acceptable to ‘starve and dehydrate’ a relative to death. This meant that very few (only two of our initial 51 interviewees) had been involved in an application to the courts for withdrawal of ANH. Since we have a specific research interest in (i) how family members experience court cases and (ii) how family members experience the death of a relative from ANH-withdrawal, we have subsequently recruited families who could report on these experiences.

The analysis presented in this article, then, draws on in-depth narrative interviews (around 25 h of recorded data) with 10 people from five different families whose relative was the subject of an application to the court for ANH-withdrawal. Interviews were recorded (sometimes just audio, sometimes both audio and video), transcribed orthographically and subjected to thematic analysis.^18^ Video clips from some of the interviews are available online at http://www.healthtalk.org.

Our sample size represents probably around 5% of all families in England and Wales who have ever been involved in applications of this type that reach court.^iii^ Two families were involved in the court process when we first interviewed them and provided us with copies of court bundles and associated correspondence, and invited us to attend court hearings. The other three families were interviewed some years after their relative had died. Interviewees included the parents, adult children, siblings and in-laws of the patient and several were interviewed more than once (eg, before and after their relative's death). We avoid giving further details due to issues of confidentiality.

In presenting our findings, we have taken care to maximise confidentiality while protecting, as far as possible, the integrity of our data. Some interviewees wanted to be anonymous, but others were willing to be identified publicly, including in filmed extracts from their interviews posted online (http://www.healthtalk.org/peoples-experiences/nerves-brain/family-experiences-vegetative-and-minimally-conscious-states/topics). However, even these interviewees sometimes wanted particular *parts* of their interviews to be anonymised. Additionally, although the patients referred to in these interviews are dead, Court of Protection reporting restrictions may still apply and we—and our interviewees—were concerned to protect the identities of health care professionals and care homes involved, as well as other family members. We have developed a set of strategies for managing these anonymising challenges, including avoidance of details duplicating those in court transcripts and omission of pseudonyms in some cases to prevent jigsaw identification.^19^

## Findings

Our key finding is that although families did receive some reassurance from the procedures involved in judicial approval, it is an oversimplification to point simply to the ‘reassurance’ provided. The process also had the opposite effect, causing distress and anxiety. Moreover, families were not simply passive recipients of reassurance but also active agents in the court application process. All five families had taken the initiative in launching the application process and challenging delays; they were involved in the process of preparing for the court hearing and they saw themselves as behaving courageously on behalf of their relative in actively supporting the decision to withdraw ANH.

### Reassurance: the court as ‘comfort’ and ‘shield’

Reassurance is a slightly patronising term, perhaps reflecting the medical and legal paternalism of the 1990s, which treats families as the passive beneficiaries of the court. Our analysis shows that it does, however, capture some aspects of family experience: in particular, the court was a ‘comfort’ and a ‘shield’ for relatives who felt burdened by the ‘momentous’ life-and-death decision concerning their relative.

All interviewees recognised the huge significance and gravity of the decision to withdraw ANH. They wanted the decision-making process to be managed with care, professional oversight and ‘in a balanced way’ (Harry) with ‘time for reflection’ and ‘safeguards’ (Helen). As Tim explained:

These are momentous decisions that need to be done by a disinterested, authoritative and experienced party […] it's only right that momentous decisions like that are made in a forum which […] steps back a bit from the individual hospitals and individual practitioners—just to make sure that the decisions are taken as dispassionately as possible.

Interviewees felt they bore a heavy burden of responsibility for ANH-withdrawal—even when (as for both the speakers below) it was actually the Trust that brought the application to court. One very positive effect of the court hearing was to share that burden:

To know actually that someone else who isn't emotionally involved is looking dispassionately at those facts and coming to the same conclusion on a humanitarian basis is actually very comforting. (Josie)

If the Court of Protection wasn't there to say “Well we are making the ultimate decision, […]” I would always feel that it was me who'd actually chosen to do it. And that would be hard […] to live with that—almost feeling that you'd sentenced them to death, however much they wanted it. So the Court of Protection has shielded me from that experience, which is good. (Hannah)

These families point to positive aspects of the requirement for a court application for ANH-withdrawal, which we would want to retain in any alternative procedure advocated.

### Initiating the process and challenging delays

Even while recognising the value of the oversight provided by the court, families were often frustrated and dismayed by the experience overall—and the requirement for judicial approval had not shielded them from responsibility for initiating the process of ANH-withdrawal (and the responsibility associated with that). The process by which their relative's case finally reached court was one they experienced as disjointed and ineffective: they struggled with unnecessary delays in a procedure administered by people who often did not seem to understand the system they were supposed to be operating.

In all five cases, it was family members (not healthcare teams) who initiated discussion about withdrawal of ANH. One application was made by the family, another had been started by the family but the Trust subsequently agreed to take it over and the remainder were launched by the Trusts at the prompting of the families—who actively pursued the application process when it seemed to falter or stall. Approval was eventually granted in connection with four of the five patients represented here: in each case, ANH was then withdrawn. In the fifth case, the patient died from other causes before the process was complete.

Although all five families had been involved in best interests consultations with clinicians concerning ceilings of care for their relative (eg, no cardiopulmonary resuscitation; no aggressive treatment of infections), some had been unaware that it might also be possible to withdraw the feeding tube. One interviewee learnt about this possibility from a newspaper article. Another was told about it by her therapist. For both, this came some years after their relative's injuries.

I said “You know, there are times, when I just wish he [PVS son] would go to sleep and never wake up”. And she said “Well if that’s what you truly, truly want, it can be achieved”. And I sort of looked at her and said “Oh yes, and how?” And she said “Seriously, there is a process” and said “what you need to do is write to your Area Health Authority and ask them if they’re prepared to work with you to put a legal case together.” […] [*Interviewer: And this had never been raised with you by the GP or the consultant or the nursing home?]* No. Absolutely not. No no.

In another family, ‘lack of communication’ both about the patient's diagnosis and prognosis and about the option of withdrawing tube feeding left the family “second guessing at the future, at the state of Mum, where she was going”. They would have liked “to know it [withdrawal] was an option […] You know, we were years down the line, not even knowing.”^iv^ These families felt very strongly that they should not have been put in the position of discovering for themselves that ANH-withdrawal was a lawful possibility and then having to initiate discussions with clinicians.

I don't think the onus should be on the families to raise the matter of whether the person […] would be better off to be actually dead because that's really troubling to have to even come to that conclusion, I think, about someone you love.^v^

We shouldn't have had to raise it. The consultant said it was normal for the family to come to raise it. Dr Smith said “oh the family need to come round to this way of thinking”. That's rubbish. The family need information which is clear if they're going to come round to anything.

Although one family felt supported by the healthcare team from the moment of initiating the discussion about withdrawal (“I can’t commend them enough… They have really tried to take everything into consideration and they were very keen to follow [the patient's] wishes”), the other four families were dismayed that once they had raised the issue of ANH-withdrawal there seemed to be no system in place for taking their concerns forward. Helen said that it took 2 years between notifying the health authority that she believed ANH was not in her son's best interests and the formal application to the courts.

A month later they [the health authority] hadn’t even acknowledged the letter. So I wrote to them again and said, “Look, I know this is a big ask, but could you tell me how far you've got. […] They sent me a letter to say […] “You realise this is quite difficult. We’ll pass the matter on to our legal department and when we get some feedback from them we’ll pass it on to you.” So nine months later nothing had happened.

Two families believed that religious values of healthcare staff may have accounted for the problems. Lack of knowledge from professionals about the process was also commonly cited:

The consultant had never done anything like this before and neither had the Primary Care Trust. It was a massive learning curve until we got hold of [the lawyer] and then she drove it. But the organisations doing the caring didn't have a clue what to do.

We were the victims of no system. There is no system to deal with it. People within the profession appear to be ignorant of what the appropriate pathways can be and therefore are not in a position to give advice to relatives.^vi^

Delays were also occasioned by disputes about who should fund the application:

When my sister was in hospital it was the hospital Trust that were responsible […]. Then she went into a community care situation by going into [Care Home] and then the responsibility changed to the Primary Care Trust. And it took again another six months for them to argue the toss about who’s responsible. […] The PCT at one stage said “You'll have to foot the bill if you want to take this forward”. […] I was thinking, “How will I do that? I'd have to remortgage the house”. Then they came back and said, “No, we'll do it.” (Harry)

Patients’ medical records were often dispersed across several different hospitals, rehabilitation centres and care homes and even getting the paperwork together could take several months. Expert reports required for the application were also a cause of delay: “Dr X was very good—he got the report to us so quickly. But Dr Y we had to wait and wait and wait for the report.” Interviewees felt as though they had been forced into a situation in which they were ‘fighting’ and ‘bullying’ medical professionals. One described the lengths he felt another family member had needed to go to in order to ensure progress was made:

[He] never got off the email to them and just badgered, badgered, badgered them. But had he not done that—the poor people at the PCT!—had he not done that, badgered and emailed and chased this up, I think it would have been a lot longer. Whether we would be there now I don't know.

There was also a significant delay between submitting the application to the court and the judicial decision—ranging, for these five families, between 4 and 14 months.

The cumulative effect of these different sources of delay (delay before families realised that ANH-withdrawal was possible; delay from that point to making the application; delay from the application to the court hearing) could be quite substantial. For example, one family began to believe that it might be in the best interests of their relative to be allowed to die about 6 months after the precipitating incident (a stroke) and the family only came to understand that withdrawing ANH was a possibility two and a half years later. They raised it with the Trust at a first best interests meeting convened for this purpose—at which it was agreed that ANH-withdrawal was indeed in the patient's best interests. It took more than another year to prepare the legal case and submit the application—which was followed by a further 5-month delay before the court hearing. The patient's brother commented: “my sister would have been horrified that it took four and a half years to attain a compassionate and dignified end to her life”. Another family who describe the process as ‘hideously slow’ formally requested ANH-withdrawal in letters prepared for a best interests meeting held 14 months after the precipitating event (a car accident). It took a further 7 months before the Trust submitted an application to the court and another 4 months before the case was heard (and further medical tests requested). Nearly 4 years after the precipitating event, the patient's son found these further delays ‘unforgivable’.

Overall, family members experienced the process of pursuing ANH-withdrawal ‘traumatic’, ‘emotionally and mentally draining’ and ‘like banging our heads against a brick wall’. They felt angry and distressed at the amount of time and effort they had to put into this (“especially after you've been through so much”). One interviewee said: “It's just been months and months of agony and frustration and the hurt goes on and on.”

### Active involvement in the court process

Far from simply being the passive recipients of reassurance, interviewees were fully involved in preparing for the court hearing (eg, all families wrote statements supporting withdrawal to be presented to the courts). The court hearing itself (which families may—but are not required to—attend) marks the culmination of months or even years of work by families to get to this point. Four of the five families believed that without their active initiation and involvement the court case for ANH-withdrawal would never have happened. Interviewees commented that it would have been far easier to cede responsibility to the care homes, Trusts and medical professionals and just let ANH continue rather than to disturb the status quo by insisting on (and actively pursuing) an application to the courts. A member of one family commented:

We were proud of us, in a way, that we had the guts to get up and speak for her. […] But we did it because we care and we thought it was a braver thing to do, to go through the court process. (Olivia)

A member of another family expressed a very similar point of view:

I think it's quite brave to say, “Well actually, let's be responsible about this and withdraw nutrition and hydration,” and take the responsibility. […] We […] didn't just let things carry on […] we had the courage to actually make a decision, with the court's approval, that we should withdraw. (Miggy)

Every one of these interviewees believed that ANH had been delivered for too long, and that their relative should have been allowed to be ‘at peace’ much sooner and they were proud of their role as a family in creating the conditions under which that could (finally) happen.

Families were generally positive about the way the case was handled in court, in particular they appreciated the comments of judges—clearly designed with the feelings of family members in mind—about the care and courage shown by the family and the appropriateness of allowing death. Seven members of one family attended court (accompanied by one of the researchers) and were impressed and moved by the gravity of the occasion, the seriousness with which the best interests of their relative were discussed and the sensitivity of the judge. At a family meal afterwards in a pavement café, they toasted their relative and celebrated the fact that he would soon be at peace (“it was a lovely day!”). Members of another family attended court via a video-link in a solicitor's conference room in another city—with “tea and biscuits … the loos were on hand… there were boxes of tissues… and the judge and the QC (Queen's Council) handled it beautifully”. An interviewee from a different family read the judgement for the first time and recalled “how kind everybody was at the court” and found the judgement “rather beautifully expressed”.^vii^

Despite these favourable comments about the court hearing itself, the many months of preparation in the run-up to the hearing caused a great deal of anxiety. In one family, two close relatives who wanted to attend the directions hearing were unable to do so because, after months of delay, it was announced at 3 days notice while they were abroad. Some expressed concerns about what they experienced as the ‘adversarial’ nature of the process. The widely publicised case of *W v M*^20^ in which the court refused a family's application for ANH-withdrawal from a minimally conscious patient on the basis of a ‘sanctity of life’ argument added to these concerns. Several interviewees assumed that the expert clinician appointed by the Official Solicitor was likely to argue against ANH-withdrawal: “specialists came down [to examine the patient]—obviously one ‘For’, one to be ‘Against’” (David). In recent cases, as some families were aware, a great deal of emphasis has been placed on accurate diagnosis as a prerequisite to making a decision about withdrawal. Anxiety about the possible consequences if their apparently ‘vegetative’ relative were to be re-diagnosed as ‘minimally conscious’ (as happened in *W v M*^20^) led to additional stress for families who were committed to ensuring a ‘dignified end’, and were not interested in the niceties of diagnostic categories because this did not change their view of what their relative would have wanted. Confronted with the suggestion that his mother might be MCS rather than PVS, her son responded: “it didn't make any difference to me; to me it was academics splitting hairs. And missing the point.” From this perspective, improvements in diagnostic technologies (eg, fMRI scanning^18^) that might result in patients who were previously labelled ‘vegetative’ now being labelled ‘MCS’, and denied ANH-withdrawal as a consequence, are seriously problematic: far from safeguarding their relative, these revised diagnoses can lead to continued administration of treatment which (in the view of family members) is not in the patient's best interests. The key issue, for these families, was that their relative would not want to be alive in their current condition—however, that condition is formally labelled and categorised.

Families who wanted to attend court were anxious about what it would be like—most had no previous experience of being in a courtroom. One interviewee who “really didn't know what to expect” asked the senior clinician who had assessed her relative:

At first I was thinking, “Oh my God, are they going to be interrogating me?”…Professor Z said, “No … it'd be highly unlikely they’d ask a member of the family to stand up and answer questions…” […] He said, “you know, you don't have to be there”.

She was also concerned that “all the [news]papers would go to town on this case […] and you might get […] people who were very much against it and would call you a murderer for agreeing to it”. Despite their largely favourable experiences of the court hearing itself, several interviewees agreed that ‘people in authority’ (such as clinicians and ethics committees) could serve the same function as the court and some remained dubious about the requirement for court approval. Members of one family (both parents and the sister of the patient) were unanimous on this, believing that “the court is the wrong place for this sort of thing”.

I think it would be much better if you could just decide it with the clinicians and do without the court case. […] I didn't actually mind attending court. It was just such an incredibly long process […] It seems more sensible that it could just be decided around a table […] I mean presumably you don't involve a court in ‘do not resuscitate’. (Miggy)

## Conclusion

Only a tiny minority of families with PVS relatives are involved in court applications for ANH-withdrawal. These interviewees are exceptional people. They have overcome enormous barriers to ANH-withdrawal, including the complexities of navigating the healthcare and legal systems, and lack of knowledge, inertia or opposition from heathcare professionals.

In our broader sample, some interviewees (especially those within a couple of years of the precipitating incident) believed that ongoing ANH was appropriate for their relative. These families sometimes reported being reassured by the legal barriers placed in the way of ANH-removal from PVS patients. One interviewee, for example, consulted a lawyer about her concern that the hospital would withdraw her husband's treatment and was comforted when informed about “how hard it is to turn off someone’s feed. […] you have to go to the High Court and it’s a very big thing to have done” (Felicity). Other interviewees had come to believe that their PVS relative would rather not be kept alive but could not accept ANH-withdrawal as an ethical option, believing it to be ‘cruel’ or ‘barbaric’.^14^ The special concern that these families feel about ANH-withdrawal is reinforced by the legal treatment of ANH-withdrawal as qualitatively different from other treatment withdrawals, such that it alone among treatment withdrawals requires judicial approval. Additionally, some were intimidated at the idea of pursuing a court case and said that they would feel judged or ‘on trial’ themselves. For our broader sample, then, the effect of Practice Direction 9E is to act as a deterrent to ANH-withdrawal from PVS patients. This is clearly reassuring for those who do not want ANH withdrawn, but imposes an additional barrier for those who believe that ANH-withdrawal might be in their relative's best interests. It tilts the balance in favour of continuing treatment, irrespective of the best interests of the patient.

So where does these empirical data leave us in relation to the claim that court approval for withdrawing ANH in PVS cases is necessary in part for the reassurance of patients’ families? Our analysis shows that it does capture some aspects of family experience (especially in relation to sharing the burden of decision making) but renders other aspects invisible—particularly families’ initiation of, and active engagement in, the process. What these families were struggling for was so much more than ‘reassurance’ for themselves. They were defending values like ‘human dignity’, ‘respect for [the patient's] wishes, and her as a person’ and ‘a peaceful death’. They were willing to bear responsibility for the ethics of their actions—but did feel vindicated and supported by the court's decision. On the positive side, then, experience of reassurance was certainly reported; on the negative side, this came at the cost of a lengthy and frustrating process. Especially in the context of other criticisms of the court application procedure (its expense, the delay it imposes and the legal anomaly it represents^9^), there is a strong case for considering whether the benefits if offers families could be provided through some alternative less expensive and more streamlined procedure.

Finally, insofar as the requirement for judicial approval is based, in part, on a perceived need to provide reassurance for families, it is somewhat misplaced. It is the patient (and not the family) that the court is there to protect. We do not believe that ‘reassuring’ patients’ families (and we have shown that the evidence on this is at best mixed) should take priority over acting in the best interests of the patient. Delivery of futile care is recognised as a widespread problem in clinical practice, yet in the five cases examined here patients were treated with ANH for many months after it had been (unanimously) determined at best interests meetings that this treatment was not in the patients’ best interests. Two recent Court of Protection cases concerned PVS patients for whom ANH had been provided for 4 years^viii^ and 9 years,^ix^ respectively: in both cases, the family, the Clinical Commissioning Groups and the Official Solicitor were in agreement that withdrawal was in the patient's best interests and in both the judges declared withdrawal to be lawful: but neither conveyed any concern about the inappropriate medical treatment meted out to these patients over such long periods of time.

On the basis of the reports from these five families, set alongside other criticisms of the current procedure,^7–10^ we believe that we urgently need to develop other more efficient, just and humane mechanisms for reviewing best interests decisions about ANH-withdrawal from these patients.

One possibility would be to treat ANH-withdrawal from PVS patients just like ANH-withdrawal from all other patients. This would mean in accordance with common law and professional guidelines^5^ ^6^ ^21^ that clinicians would be able lawfully to withdraw ANH without recourse to the courts if they determined that ANH was not in the patient's best interests (and if this was not contested). It has already been argued that there is no legal rationale for treating PVS (and MCS) patients differently from all others:^9–11^ equality of treatment under the law would recognise their right to equal protection from futile, burdensome and unwanted treatment that is not in their best interests. For patients’ families, this would remove one important source of the delays during which inappropriate treatment continued to be administered to their relative, and would have allowed the patient to be ‘at peace’ much sooner. Families would no longer have to endure (what some experienced as) the continued medical ‘torture’ of their relative over the course of additional months or years of waiting for a court hearing, nor face the steep learning curve, stress and anxiety involved in engaging with an ‘adversarial’ court process. We also suggest, on the basis of our broader data set, that more families would be able to accept ANH-withdrawal if the deterrent effect of a court application were removed—especially given the special significance it adds to the already symbolically powerful meanings of withdrawing nutrition and hydration.

Set against these benefits would be the loss of ‘therapeutic jurisprudence’.^9^ To preserve these benefits, healthcare teams making best interests decisions not to continue ANH-treatment would need to ensure (as they should do currently^22^) that family members know that the decision is not theirs to make, that the burden of responsibility lies with the clinical team and that everyone involves recognises the immense gravity of the decision to allow death. Excellent palliative care of the type approved by the courts would also need to be ensured, as well as considering how to address what families often experience as major differences between withdrawal from patients who are clearly dying irrespective of ANH-withdrawal and those in PVS/MCS who could be maintained for years or decades. Finally, simply abolishing the requirement for a court hearing will not address the broader problems faced by these patients and their families, which relate to the (sometimes total) absence of best interest consultations, default presumptions of continued treatment and decision making based on criteria other than the best interests of the patient.^11^ ^14^ ^15^

If abolishing the requirement for judicial approval seems a step too far, there are steps that could be taken to speed up the court process. These cases are often not seen as ‘urgent’—compared, for example, with life-threatening situations such as disputes about emergency surgery which can be heard by the courts within days or even hours. When a PVS patient's feeding tube perishes or becomes dislodged and the need for replacing it is challenged, cases can reach court very quickly (eg, in Re D (Medical Treatment) [1998] 1 FLR 411 within 3 days). But so long as ANH is continuing then, despite the existence of a best interests decision (by clinicians in consultation with family and carers) that it should not be, there is no apparent urgency to protect patients against potential assault (ie, invasive treatment not in their best interests). Shifting priorities in the judiciary to recognise the importance of hearing cases where ANH treatment is continuing just as rapidly as when it is not would benefit patients and their families—and since there are relatively few such cases this should not impose too much of a burden on the system.

Other suggestions for speeding up the court process include permitting consideration of applications concerning PVS patients by Court of Protection judges at District Judge and Circuit Judge level (not only by the President of the Court of Protection or High Court judges nominated by him) and requiring only written (rather than written and oral) evidence.^23^ The latter procedure could be modelled on *Re X*^24^ which was introduced to streamline Deprivation of Liberty cases and would involve filing specified documents (see Annex A of ref. 24), which would include demonstrated compliance with relevant guidelines and written evidence from family and carers. The evidence could include an independent second medical opinion and evidence of the patient's likely wishes and the steps taken to identify those wishes. The decision could then be made on the basis of written evidence unless there was a disagreement concerning diagnosis, in which case an oral hearing would be required.

In summary, the combined evidence (to which we have contributed here the experience of family members) provides strong support for the claim that the current requirements and procedures for approving ANH-withdrawal from PVS patients are not working. They are expensive, cumbersome, protracted and unclear to people caught up in them. They add to, rather than relieve, human suffering. They are not serving the best interests of patients (or their families). As a society, we can and must do better than this.

## Footnotes

i Lord Keith of Kinkell in *Bland*, n.34.

ii *Airedale NHS Trust* v *Bland* [1993] AC 789, per Lord Goff at 873. Lord Goff in the House of Lords was approving a ruling by Sir Thomas Bingham in the Court of Appeal, who in turn was approving the ruling of Sir Stephen Brown P at first instance. Other components of the rationale included protecting patients, protecting doctors and reassuring the public.

iii There are just over 50 reported cases but many judgements in the Court of Protection are never reported and we know that some decisions about ANH-withdrawal from PVS patients have been among them, including one in the current sample. Our best estimate is that 100+ such cases have been heard.

iv Read more: http://www.healthtalk.org/peoples-experiences/nerves-brain/familyexperiences-vegetative-and-minimally-conscious-states/family--experiences-decision-making#ixzz3RuNfy9Ds

v Read more and see longer clips of family members describing their experience at: http://www.healthtalk.org/peoples-experiences/nerves-brain/family-experiences-vegetative-and-minimally-conscious-states/family-experiences-applying-court-treatment-withdrawal#ixzz3RH2WjEF4

vi http://www.healthtalk.org/peoples-experiences/nerves-brain/family-experiences-vegetative-and-minimally-conscious-states/gunars-and-margaret

vii http://www.healthtalk.org/peoples-experiences/nerves-brain/family-experiences-vegetative-and-minimally-conscious-states/family-experiences-applying-court-treatment-withdrawal

viii *NHS Trust v AW* [2013] EWHC 78 (COP).

ix *Gloucestershire Clinical Commissioning Group v AB* [2014] EWCOP 49.
